# Pareto Optimized Adaptive Learning with Transposed Convolution for Image Fusion Alzheimer’s Disease Classification

**DOI:** 10.3390/brainsci13071045

**Published:** 2023-07-08

**Authors:** Modupe Odusami, Rytis Maskeliūnas, Robertas Damaševičius

**Affiliations:** 1 Faculty of Informatics, Kaunas University of Technology, 51368 Kaunas, Lithuania; 2 Faculty of Applied Mathematics, Silesian University of Technology, 44-100 Gliwice, Poland

**Keywords:** deep learning, pareto optimization, image fusion, fusion weights, MRI, multimodal imaging, PET

## Abstract

Alzheimer’s disease (AD) is a neurological condition that gradually weakens the brain and impairs cognition and memory. Multimodal imaging techniques have become increasingly important in the diagnosis of AD because they can help monitor disease progression over time by providing a more complete picture of the changes in the brain that occur over time in AD. Medical image fusion is crucial in that it combines data from various image modalities into a single, better-understood output. The present study explores the feasibility of employing Pareto optimized deep learning methodologies to integrate Magnetic Resonance Imaging (MRI) and Positron Emission Tomography (PET) images through the utilization of pre-existing models, namely the Visual Geometry Group (VGG) 11, VGG16, and VGG19 architectures. Morphological operations are carried out on MRI and PET images using Analyze 14.0 software and after which PET images are manipulated for the desired angle of alignment with MRI image using GNU Image Manipulation Program (GIMP). To enhance the network’s performance, transposed convolution layer is incorporated into the previously extracted feature maps before image fusion. This process generates feature maps and fusion weights that facilitate the fusion process. This investigation concerns the assessment of the efficacy of three VGG models in capturing significant features from the MRI and PET data. The hyperparameters of the models are tuned using Pareto optimization. The models’ performance is evaluated on the ADNI dataset utilizing the Structure Similarity Index Method (SSIM), Peak Signal-to-Noise Ratio (PSNR), Mean-Square Error (MSE), and Entropy (E). Experimental results show that VGG19 outperforms VGG16 and VGG11 with an average of 0.668, 0.802, and 0.664 SSIM for CN, AD, and MCI stages from ADNI (MRI modality) respectively. Likewise, an average of 0.669, 0.815, and 0.660 SSIM for CN, AD, and MCI stages from ADNI (PET modality) respectively.

## 1. Introduction

### Background

Millions of people suffer from the degenerative neurological condition known as AD worldwide. AD impairs cognition and memory, thereby weakening the brain gradually. To effectively treat and control AD, it is essential to get an accurate and timely diagnosis. Diagnosis of AD using neuroimaging techniques has become one of the most reliable ways of diagnosing Alzheimer’s disease, because of the rising growth of neuroimaging technologies [1,2]. The use of multimodal imaging methods to diagnose AD, such as PET and MRI, has also grown in usage [3,4,5,6]. These imaging methods can provide a more holistic view of the dynamic alterations that occur in the brain over time in AD  [7], assisting in the understanding of the disease’s pathophysiology. Considerable research has been done on multimodal neuroimaging data by using information from the different modalities at different fusion levels [8,9]. Diagnosis of AD at the prodromal stage was achieved by combining features from MRI and PET images using an adaptive similarity matrix to obtain intrinsic similarity shared across sMRI and PET data [10]. Supplementary information provided by MRI and PET based on consistent metric constraints was used to achieve higher classification accuracy for AD classification [11]. In addition, a cascaded convolutional neural network (CNN) was developed to autonomously comprehend the multimodal characteristics of MRI and PET brain images to classify AD [12]. Nonetheless, the clinical comprehension of brain abnormalities through learned features is impeded by the inadequacy of clinical data available to identify associated patterns. Sparse multi-task learning and discarding uninformative features from MRI and PET were iteratively performed to achieve optimal feature sets for AD classification [13]. A sparse learning method was used to harness features from MRI and PET to jointly predict the clinical scores and classify AD stages  [14]. A sparse interpretable Graph Convolutional Network was utilized to identify important node features for AD classification from multimodal imaging of MRI and PET images [15]. Although some of the sparse learning methods gave an impressive results in AD classification, the method is very complicated and requires extensive high computational resources. Apart from this, the selected fused features may be ineffective for modeling complex brain patterns [16]. Some of the fusion techniques can provide fused information that enables more comprehensively structural and functional information for AD diagnosis. However, several assumptions have to be made, and this may not provide the optimal set for AD diagnosis  [17], and some of the features chosen may be insufficient to represent the underlying information from the original data [3,18].

To provide more accurate and informative output, medical imaging fusion  [19,20], a specific algorithm to combine two or more images into a new image has been utilized in most of the existing studies in diagnosing AD [21]. Numerous studies have focused on using multi-scale-based transforms to improve fusion effects in the field of AD diagnosis [22]. These studies have specifically targeted the improvement of fusion effects in AD diagnosis research by employing multi-scale-based transforms, to enhance fusion effects in AD diagnosis.Information from MRI and PET images is fused based on Discrete Wavelet Transform (DWT) by capturing the frequency and location information, and transfer learning is used to optimize the fusion process [8]. While this fusion approach improved the information obtained from MRI and PET imaging modalities, interpreting the fused images proved difficult. The demon algorithm and DWT were utilized to attain an optimal fusion of MRI and PET [22]. This method combined the anatomical information provided by MRI with the functional and metabolic data obtained from PET. The Demon algorithm enabled robust registration for proper alignment, while DWT provided valuable insights into both global and local features of MRI and PET data. The demon algorithm, on the other hand, is dependent on accurate image registration, which can be difficult in the presence of anatomical variations [23,24].

Two-dimensional Fourier Transform (FT) and DWT were used in the fusion process, which combined MRI and PET images. This method used Fourier analysis and wavelet-based decomposition to combine spatial and spectral information from both imaging modalities. The resulting image was reconstructed using the inverse FT and inverse DWT [25]. A novel algorithm based on Undecimated DWT was used to effectively fuse MRI image and SPECT image for AD diagnosis [26], the low-frequency band coefficients are fused through the application of the maximum selection rule, while the coefficients of the high-frequency bandis subjected to modified spatial frequency. A parameter adaptive Pulse-Coupled Neural Network (PCNN) is utilized to fuse the salient complementary details and corresponding pseudo-color from MRI and PET images [27]. This method effectively combines information from MRI and PET, however, some of the objective performances needs improvement. Non-subsampled Shearlet Transform (NSST) coupled with simplified PCNN is utilized for combining MRI and PET [28]. This method improves the spatial resolution of the fused images, which is crucial forthe accurate diagnosis of AD. Although the method provided high-quality fused images, there is a need for more improvement in the objective performance of the fused image. Furthermore, a novel fusion approach using NC Contourlet Transform (NSCT) coupled with two different fusion rules is proposed for MRI and PET fusion  [29]. The prevalent methodology for image fusion in the transform domain entails the conversion of the source image into sub-bands of frequency, followed by the fusion of sub-bands based on frequency coefficients.

Finally, an inverse transform is applied to reconstruct the merged image. The utilization of the transform domain-based technique offers various benefits such as a well-defined structure and minimal distortion, however, this method suffers from noise during the fusion process, thereby producing artifacts around edges that can deteriorate the information in the fused image  [30,31,32]. These artifacts are caused by imagetransformation and the fusion rule for the decision feature map [33]. This feature map is created by measuring activity levels and then assigning weights to them [34]. However, the activity level measurements are not resistant to noise and misregistration, and their design is difficult without compromising algorithm performance [35]. As a result, there is an increasing interest in creating more robust and efficient activity-level measurement methods that can deal with noisy and misregistered images while maintaining a high-performance level [36,37]. The motivation for this research is to provide better accuracy and reliability of image fusion techniques, particularly in the context of AD classification, where accurate diagnosis is critical for early diagnosis. This study addresses this problem by using deep learning model networks to create a weight map and activity levels measure model that is both robust and efficient [35].

In this research paper, the potential of deep learning techniques for fusing MRI and PET images using pre-trained models such as VGG11, VGG16, and our own, Pareto optimized variant of VGG19 architecture is investigated. This research entails an examination of the efficacy of the three VGG models in capturing significant features from the fused MRI and PET data. A transposed convolution layer that takes the output from the original convolution layer is utilized to modify the VGG models. The transposed convolution restored the size of the feature map, thereby preserving spatial information and enhancing the representation of the fused image. The processing steps utilized in this research provide Structural and functional property alignment. The model that exhibits the most optimal performance is subsequently proposed for image fusion purposes.The evaluation of the models is done on the ADNI dataset using SSIM, PSNR, MSE, and E.

The main contribution of our work is summarized as follows:

- The proposed model examined the effectiveness of the pareto optimized VGG model vs. traditional VGG variants in extracting significant features from MRI and PET data to assess how well these deep learning models can extract important features.
- Each convolution layer is examined to know the layer that produces the feature map with the best image quality.
- To enhance the effectiveness of VGG models, a pareto optimization and transposed convolution layer has been incorporated to enable the restoration of the feature map’s proportions while concurrently preserving spatial information.
- The incorporation of transposed convolution enhances the representation of the fused image, leading to an overall improvement in the effectiveness of the models.

The present paper is structured as follows. In Section 2, the relevant theories utilized in our proposed approach were explicated, along with a comprehensive account of the fusion technique. Section 3 of the paper outlines the experimental settings, while Section 4 presents the results of the study, including a comparison with previously established image fusion techniques. In conclusion, the present paper is concluded in Section 5.

## 2. Methods

**Figure 1:**
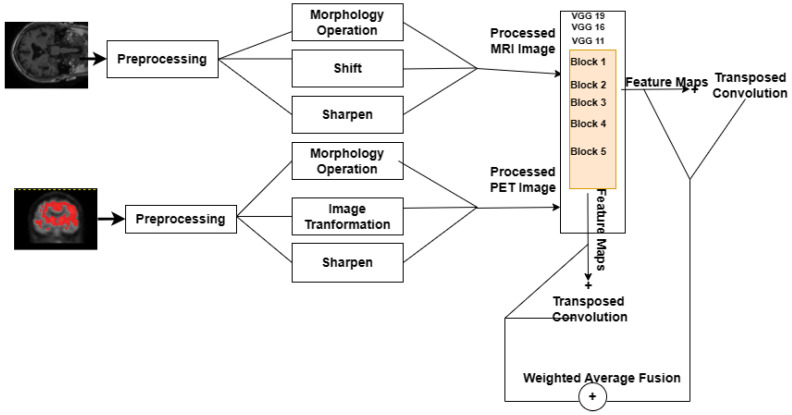
Proposed Image Fusion Framework.

In this study, the potential of deep learning techniques is investigated for fusing MRI and PET images using pre-trained models such as VGG11, VGG16, and VGG19 architecture which have demonstrated remarkable performance in several computer vision tasks. After applying some preprocessing techniques on MRI and PET images using Analyze Software (version 14.0) and Gimp Software (version 2.10.34), the next step is that the VGG network extracts deep features and generates weight maps from the preprocessed input images. The framework for the proposed imaging fusion technique is depicted in Figure 1.

### 2.1. Preprocessing of MRI and PET Images

**Figure 2:**
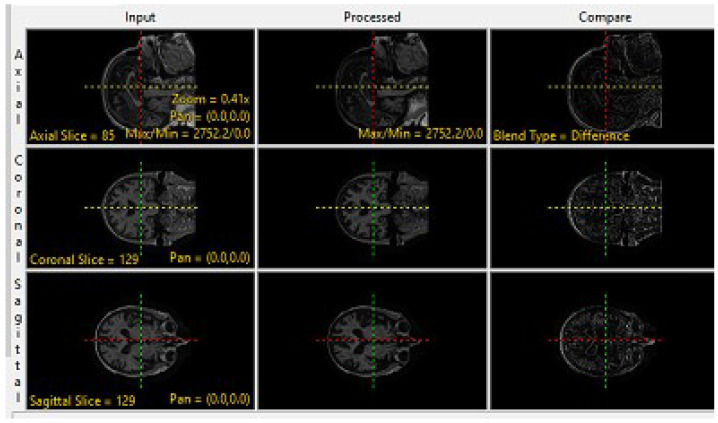
Morphologic Operation for MRI Image.

**Figure 3:**
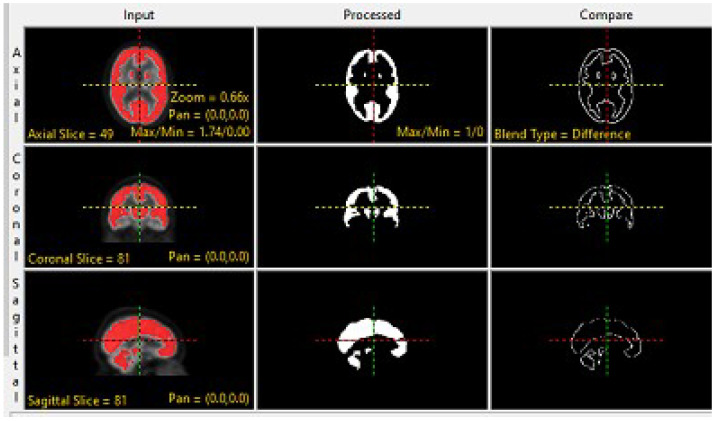
Morphologic Operation for PET Image.

The preprocessing techniques are divided into three steps: The first step includes a basic morphological operation, which involves applying basic morphological operations to the input data, such as dilation [38], for MRI images, which is a type of dilation operation that replaces each pixel in an image with the minimum value in a predefined neighborhood around it and erodes morphological operation [39], for PET images which erodes the boundaries of foreground objects in PET image while preserving their shape and size. This preliminary step aims to prepare the MRI data and PET data for further analysis by fine-tuning their structural and functional properties and reducing noise or artifacts that may interfere with subsequent processing stages. The morphology operation for both MRI and PET at coronal planes is accomplished by utilizing the analyze 14.0 software, as clearly illustrated in Figure 2 and Figure 3.

**Figure 4:**
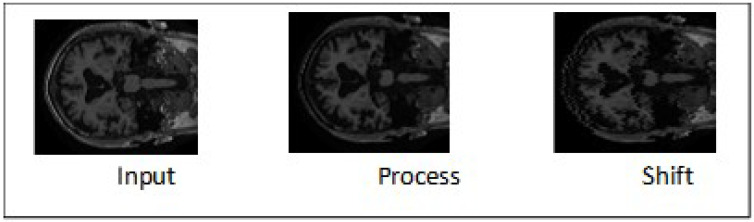
Sample Image from MCI Class (MRI).

**Figure 5:**
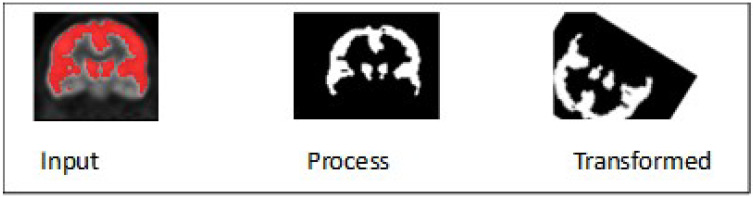
Sample Image from MCI Class (PET).

As shown in Figure 4, the second preprocessing step for an MRI image involves using a shift operation to horizontally translate the MRI image by a certain number of pixels. This shift operation enables the image to be precisely aligned and adjusted to optimize its position for further analysis and processing. The second preprocessing step for PET images, on the other hand, involves the use of the transform tool from the GIMP software. This tool allows the rotation of the PET image by a certain amount, as shown in Figure 5. Any potential misalignment or non-uniformity in the image can be corrected by rotating it, improving the accuracy and reliability of subsequent examinations and evaluations. The third step involves implementing kernel-based sharpening techniques, which aim to significantly enhance the sharpness and definition of an image’s edges and intricate details [40]. By employing this method, the MRI and PET image undergoes an adjustment that intensifies the clarity and crispness of their fine elements, resulting in a visually enhanced representation.

### 2.2. Proposed Fusion Technique of MRI and PET

Assuming a pre-trained VGG with layers *Y*, with $V_{i}$ output channels per layer *Y*. Source image *Z* is represented in Equation (1).

(1) $$Z=\left\{I_{z}\;|\;Z\in \left\{1,2,3,4,\ldots ,Z\right\}\right\}$$

A vector containing the ReLU- transformed values for each source image *z* in $F_{y}$, extracted from the image *z*-th at the layer *y*-th of the feature map *v*-th of the VGG network is represented in Equation (2).

(2) $$f_{z}^{y}=\max\left(0,F_{y}\left(I_{z}\right)\right)$$

where $F_{y}\left(\right)$ = Utilization of network layers toward the source image up to layer *y*. Max(0,.) = ReLU operation (function) to introduce nonlinearity into the output. Every feature map generated is normalized over the $V_{i}$ channels of the feature maps of layer *y*, which is represented in Equation (3).

(3) $${\bar{f}}_{z}^{y}=\sum_{v=0}^{V_{i}}{∥f_{z}^{\left(v,y\right)}∥}_{I}$$

The normalized feature maps ${\bar{f}}_{z}^{y}$ includes a measurement of the level of activity that corresponds to the input image at layer *y*. For *y* layers, feature maps are extracted for each image *z* given a set of feature maps represented in Equation (4).

(4) $${\bar{F}}_{z}=\left\{{\bar{f}}_{z}^{y}\;|\;y\in Y\right\}$$

Additionally, *Z* feature maps are utilized to create *z* weight maps for each layer y to show the contribution of each image to a given pixel. Softmax is utilized in our study to generate *z*-weight maps, and it is represented in Equation (5).

(5) $$W_{z}^{y}=\frac{e^{{\bar{f}}_{z}^{y}}}{\sum_{j=1}^{Z}{\bar{f}}_{j}^{y}}$$

Equation (5) generated a set of weights $W^{y}$ at layer, *y* represented in Equation (6).

(6) $$W^{y}=\left\{W_{z}^{y}\;|\;Z\in \left\{1,2,\ldots ,Z\right\}\right\}$$

Based on the weight map generated in Equation (6), the image fusion at layer *y* is computed as represented in Equation (7).

(7) $$I_{F}^{y}=\sum_{z=1}^{Z}\left(W_{z}^{y}I_{z}*\mathrm{TransConv}\left(I_{z}\right)\right)$$

Reconstructing the fused image from *y*-th layers involves selecting the optimal pixel. We set the weight of each layer to 1 if it contains the maximum pixel and 0 otherwise. The final fused image is represented in Equation (8).

(8) $$I_{F}^{y}=\max\left(I_{1}^{y},I_{2}^{y},\ldots ,I_{Z}^{y}\right)$$

where the *y*-th fused feature map’s max() function gives the highest pixel value possible for all layers.

### 2.3. VGG Convolutional Network Architecture

Let *X* be the input to the network, represented as a 2D array with dimensions H×W. Each element $X_{i,j}$ represents the pixel value at position (i,j).

The VGG architecture consists of a series of layers, including convolutional layers (Conv), activation functions (ReLU), and pooling layers (Pool), followed by fully connected layers (FC) for classification.

1. Convolutional Layers in VGG perform convolutional operations on the input *X* using a set of filters. Let’s denote the *k*-th Convolutional Layer as Conv_k. The output feature maps of Conv_k are denoted as $F_{k}$, with dimensions $H_{k}\times W_{k}$.

The convolution operation can be defined as:

(9) $$F_{k}\left[i,j\right]=\mathrm{ReLU}\left(\sum_{\left(m,n\right)\in A_{k}}W_{k}\left[m,n\right]\cdot X\left[i+m,j+n\right]+b_{k}\right),$$

where $A_{k}$ is the receptive field (filter size) of Conv_k, $W_{k}$ is the weight matrix, and $b_{k}$ is the bias vector associated with Conv_k. ReLU represents the Rectified Linear Unit activation function.

2. Pooling Layers in VGG perform downsampling on the feature maps. Let’s denote the *k*-th Pooling Layer as Pool_k. The output feature maps after pooling are denoted as $P_{k}$, with dimensions $H_{k}^{\prime }\times W_{k}^{\prime }$.

The pooling operation can be defined as:

(10) $$P_{k}\left[i,j\right]=\max_{\left(m,n\right)\in B_{k}}F_{k}\left[m,n\right],$$

where $B_{k}$ represents the pooling window (region) of Pool_k.

3. Fully Connected Layers in VGG take the flattened feature maps as input and produce the final classification output. Let’s denote the *k*-th Fully Connected Layer as FC_k. The output of FC_k is denoted as $O_{k}$.

The fully connected operation can be defined as:

(11) $$O_{k}=\mathrm{ReLU}\left(W_{k}\cdot O_{k-1}+b_{k}\right),$$

where $W_{k}$ is the weight matrix and $b_{k}$ is the bias vector associated with FC_k.

4. Output Layer of VGG uses a softmax activation function to produce the class probabilities. Let’s denote the output layer as Output. The final class probabilities for classification are denoted as $P_{\mathrm{class}}$.

(12) $$P_{\mathrm{class}}=\mathrm{softmax}\left(W_{\mathrm{output}}\cdot O_{L}+b_{\mathrm{output}}\right),$$

where $W_{\mathrm{output}}$ is the weight matrix and $b_{\mathrm{output}}$ is the bias vector associated with the output layer.

By stacking the convolutional layers, activation functions, pooling layers, fully connected layers, and the output layer according to the VGG architecture, we obtain the complete mathematical definition of the VGG deep neural network.

### 2.4. Transposed Convolution

The transposed convolution method is a prevalent technique employed in neural networks to increase the resolution of feature maps. It finds its application in various tasks, including image segmentation and image generation [41,42,43,44]. In Equation (7), $TransConv(I_{z})$ is applied to the input feature map and can be formally defined in Equation (9).

(13) $$TransConv\left(I_{z}\right)=TConv\left(I_{z},K,S\right)$$

where TConv = transposed convolution operation. $I_{z}$ = input feature map. *K* = transposed convolution kernel. *S* = transposed convolution operation stride.

In our proposed architecture, *K* is 3, while *S* is 1. We applied a 1 × 1 transpose layer on the input feature map as represented in Equation (10). TConv will have a shape $\left(C,H^{\prime },W^{\prime }\right)$, where $H^{\prime }$ is H+2P−K and $W^{\prime }$ is W+2P−K, and *P* is the padding size. In this study, the padding size used is 1

(14) $$TConv\left(c,h^{\prime },w^{\prime }\right)=\sum_{i}\sum_{j}\sum_{c^{\prime }}X\left(c^{\prime },h+i,w+j\right)\cdot W\left(c,i,j\right)$$

where *c* = channel index, *h*’ and $w^{\prime }$ = the spatial indices of the output feature map, *i* and *j* = indices within the kernel size, $c^{\prime }$ = channel index of the input feature map.

### 2.5. Pareto Optimality

To define VGG hyperparameter optimization using Pareto optimality, we need to establish a formal mathematical framework that relates the hyperparameters of the VGG architecture to the concept of Pareto optimality. Pareto optimality is a concept in multi-objective optimization where a solution is considered optimal if it cannot be improved in one objective without sacrificing another objective.

Let’s denote the hyperparameters of the VGG architecture as a vector $H=(H_{1},H_{2},\ldots ,H_{n})$, where $H_{i}$ represents the value of the *i*-th hyperparameter. Additionally, let’s consider *M* objective functions $f=(f_{1},f_{2},\ldots ,f_{M})$, where $f_{i}\left(H\right)$ represents the evaluation of the *i*-th objective function given the hyperparameters H.

The goal of hyperparameter optimization is to find a set of hyperparameters that maximizes or minimizes the objective functions while satisfying any constraints. In the case of Pareto optimality, we aim to find hyperparameters that achieve the best trade-off between multiple conflicting objectives.

Formally, VGG hyperparameter optimization using Pareto optimality can be defined as finding the set of hyperparameters $H^{*}$ that satisfies the following conditions:

(1) Feasibility: $H^{*}$ satisfies any constraints imposed on the hyperparameters.
(2) Pareto Optimality: There does not exist another set of hyperparameters $H^{\prime }$ such that $f_{i}\left(H^{\prime }\right)\geq f_{i}\left(H^{*}\right)$ for all *i*, with at least one strict inequality. In other words, the hyperparameters $H^{*}$ are Pareto optimal if there is no other set of hyperparameters that can achieve better values for all the objectives simultaneously.

### 2.6. Summary

**Figure 6:**
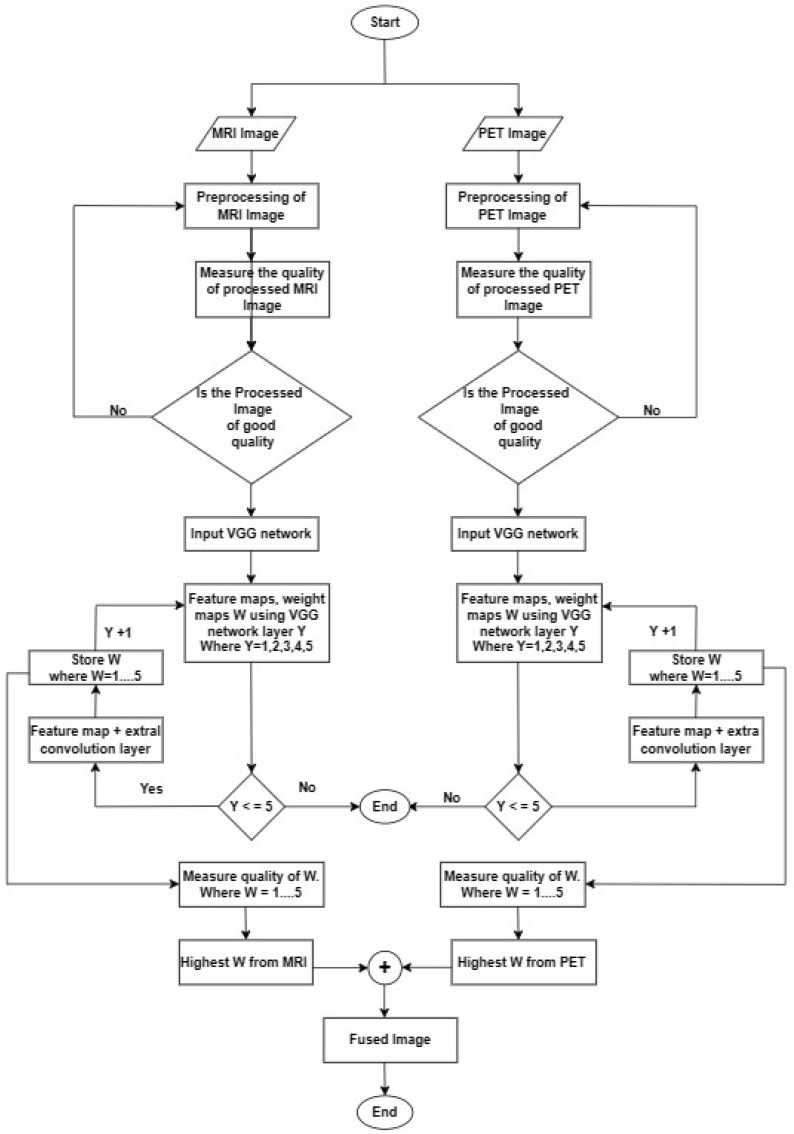
Flowchart of Proposed Method.

Figure 6 displays the equivalent flowchart of our proposed model, providing a visual representation of the sequential steps and logical connections that illustrate the underlying process and functionality of our proposed method.

## 3. Experiments

To carry out our experiments, we gathered medical images of MRI and PET modalities from the ADNI database, specifically focusing on whole brain scans for individuals with AD, cognitively normal (CN) individuals, and those with Mild Cognitive Impairment (MCI). MRI images of Magnetization Prepared-Rapid Gradient Echo (MP-RAGE) sequence with normalization are considered, as they provide excellent tissue contrast and spatial resolution, allowing for detailed visualization of the brain’s anatomical structures. PET images of average coregister with voxel size and uniform resolution are utilized to provide consistency and comparability between different images. In total, 50 images of T1 weighted MRI and 50 FDG- PET corresponding to AD, CN, and MCI stages were downloaded.In total 150 images were used to train the model.We trained the selected VGG models in order to extract feature maps and assign the necessary weight for image fusion. For this experiment, pareto optimized VGG19 [45,46], VGG16, and VGG11 pre-trained networks are used to compute the image fusion based on the feature maps at the 1st layer and compared. Multiple pooling layers in VGG reduce the resolution of the feature maps. As a result, the weight maps’ width and height are determined by the layer *Y* over which they were computed. VGG contains 5 pooling layers with large convolutional blocks and as such the fused image $I_{F}^{\prime }$ is derived from convolution block $c_{b}\in \left\{1,\ldots ,5\right\}$ as described in Equation (4). To avoid or mitigate upsampling artifacts in weight maps, depth *Y* of the convolution blocks needs to be examined critically. There is an inclusion of a transposed convolution layer to the feature map before the final fusion.Pareto optimization is implemented by introducing a parameter alpha to weight the importance of the cross-entropy loss objective and beta to weight the importance of the trainable parameters objective.By adjusting the values of alpha and beta, we explored different trade-offs between minimizing the cross-entropy loss and minimizing the number of trainable parameters.The best possible compromises between the two objectives is the optimal solution.

The present study employs objective fusion metrics, namely the structural similarity index (SSIM), peak signal to noise ratio (PSNR), and mean square error (MSE) [47], and Entropy (E) to perform quantitative assessments of the fusion of MRI and PET image fusion. SSIM quantifies the extent to which the structural information present in the input images is preserved in the resulting fusion. PSNR is a metric that measures the quality of an image by comparing the original signal or data to the noise or error introduced during image compression or distortion in the fusion process. MSE on the other hand, quantifies the level of error present in the fused image. E measures the content of information in an image. The metric denoted by “E” quantifies the amount of information present in an image. A fused image with superior performance can be indicated by higher values of PSNR, SSIM, and E, whereas a lower MSE value can suggest that the fused image has a reduced amount of error. The proposed model implementation and evaluation is performed using pytorch on NVIDIA Corporation TU116 (GeForce GTX 1660) graphic processing unit machine.

## 4. Result

**Figure 7:**
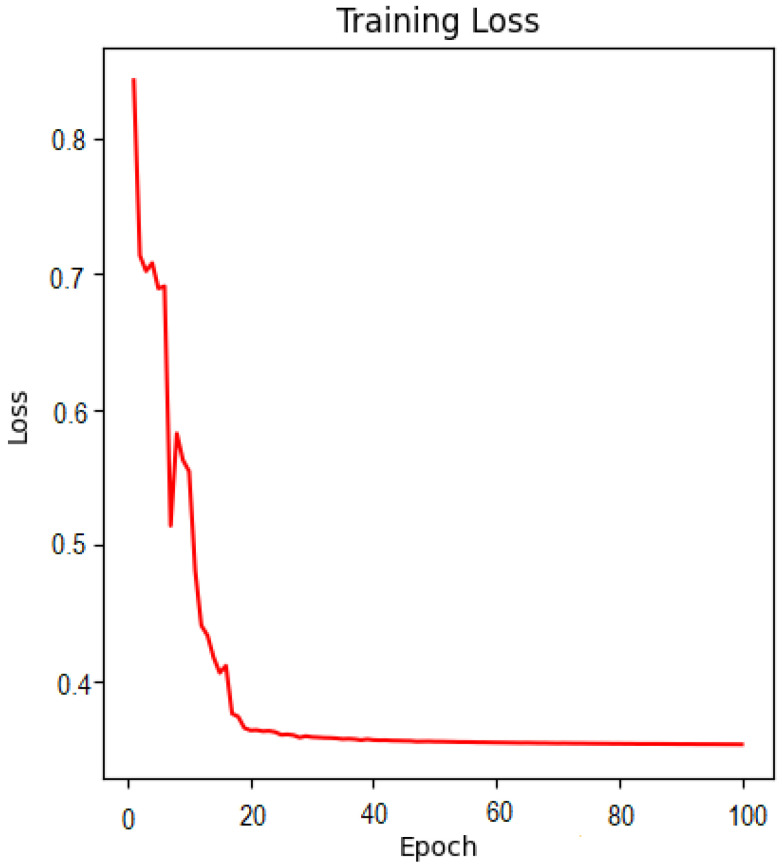
Visualization of Training Loss for our Proposed Pareto Optimized VGG19 (fragment of the first 100 epochs).

**Figure 8:**
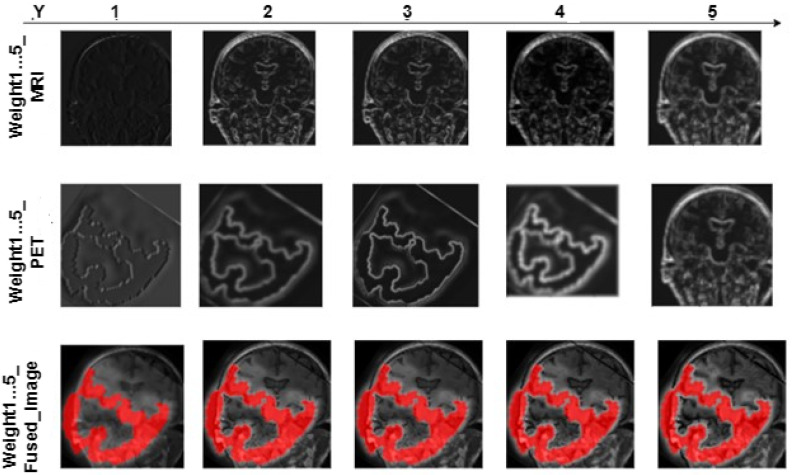
MRI-PET Fusion weights and results compared to feature depth using VGG19 without Transposed Convolution.

**Figure 9:**
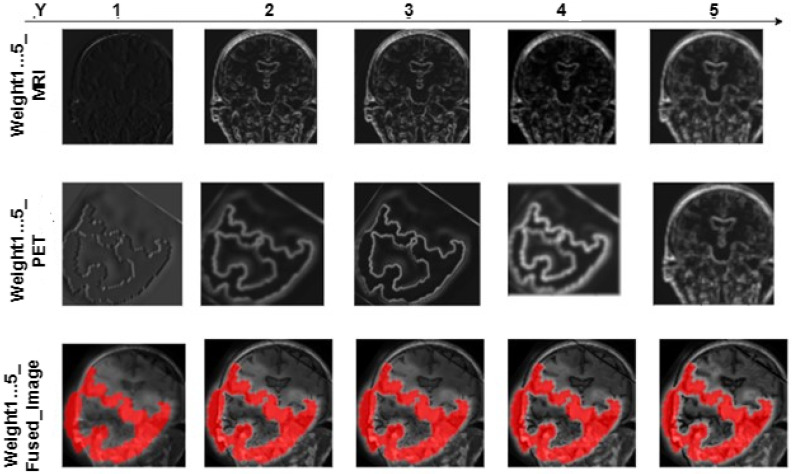
MRI-PET Fusion weights and results compared to feature depth using VGG19 with Transposed Convolution.

Table 1 shows the comparison of MRI-PET Fusion results using Pareto optimized VGG19, VGG16, and VGG11 using the adopted evaluation metrics. The given results in this section are based on the fusion of 50 MRI-PET image pairings. Figure 7 shows a loss curve for the first 50 epoch. Loss continues to drop for the small value of 0.1 over the duration of 1000 epochs. Figure 8 and Figure 9 show the progressive weight maps of MRI and PET with and without transposed convolutional layers, and the fusion results as the depth *Y* increases on the VGG19 network. These artifacts from upsampling reduce the fusion quality by introducing more unwanted noise and altering the intensity levels. Table 2 depicts the quality of the fused image to the depth of the feature consider in the weight computation with and without an transposed convolution layer with the feature maps computed on the fusion of 50 MRI-PET image pairs. Computational complexity based on Average Processing Time (APT) for each layer of extraction is also shown in Table 2 to give the impact of transposition. The result shown in Table 3 is the average value over the 50 MRI-PET image pairs. Finally, Table 3 depicts the average run time of pareto optimized VGG19 with transposition convolution and without convolution transposition.

## 5. Discussion

Table 1 shows that Pareto optimized VGG19 achieved the highest SSIM value (0.680), (0.802), and (0.664) for CN, AD, and MCI respectively in MRI modality, followed by VGG16 (0.670) for AD and VGG11 (0.560) for AD. Also, for the PET modality, VGG19 achieved the highest value across the three metrics. Similarly, VGG19 achieved the highest PSNR value (35.43 dB), (36.01 dB), and (34.31 dB) for CN, AD, and MCI respectively in MRI modality, followed by VGG16 and VGG11. Additionally, modified VGG19 achieved the lowest MSE value, followed by VGG16 and VGG11. Based on these results, Pareto optimized VGG19 outperformed the other two architectures in terms of fusion image quality. The higher values of SSIM and PSNR and the lower value of MSE indicate that our VGG19 variant generated fused images with higher similarity to the ground truth and lower distortion than VGG11 and VGG16. Because AD patients typically have more severe brain changes and atrophy than CN and MCI patients, the image fusion quality of the AD class exceeds that of the CN and MCI classes. This could result in the emergence of more prominent and recognizable brain image patterns, easing the identification of the VGG19 network.

As depth Y rises, the progressive weight maps and fusion outcomes are shown in Figure 6 and Figure 7. The weight maps exhibit undesirable upsampling artifacts due to the decreased resolution in the deeper levels [4,7,10]. The presence of these artifacts reduces fusion quality by increasing the amount of unwanted noise and causing intensity-level distortion. The mean quality of image fusion to the depth of the characteristics used for weight computation is shown in Table 3 for 50 pairs of MRI-PET images. Table 3 shows how the depth of features considered in weight computation affects the quality of image fusion. When delving deeper into the network, there is a noticeable decrease in SSIM, PSNR, MSE, and E. This means that as we move down the network layers, the quality of the output image degrades in terms of these metrics. As a result, there is an inverse relationship between network depth and the accuracy of these measures. As the network considers more complex features, the quality of the fused image deteriorates. As a result, the shallower features are better suited to the MRI-PET image fusion task. The shallower features contain more complementary information from MRI and PET. Complex features, on the other hand, do not contribute nearly as much to the final fused image quality. The results from Table 1 show that the transposed convolution layer added to the feature maps gave higher quantitative results than the conventional structure of VGG. Furthermore, it is also clear that the higher values of all the metrics are obtained at the shallow layer of the proposed VGG network. It is clear from Table 3 that the use of transposition convolution gave a higher computational complexity in terms of processing time than the conventional VGG19.The Pareto optimization technique reduced the number of parameters, and this lowered the computational complexity of our proposed model. From Table 3, our proposed pareto optimed VGG19 with transposition convolution average runtime for 50 images of MRI and PET image fusion is not as high as the one without convolution transposition, and this is due to the minimized number of trainable parameters by adjusting the value of the two objectives thereby providing best optimal solution.The objectives are to weight the importance of the cross-entropy loss objective and to weight the importance of the trainable parameters objective.

### 5.1. Comparison to Other Image Fusion Techniques

This section presents a comparison of the proposed method with existing approaches based on quantitative measurements utilized in the study. The techniques under comparison are as follows: DWT with transfer learning [12], PCNN with parameter adaptive [27], NSST coupled with PCNN [28], and NSCT [34]. DWT with transfer learning decomposed images into low and high frequency bands based on DWT, and VGG16 was used to fuse the relevant information from MRI and PET. Finally, IDWT was used to reconstruct the final fused image. PCNN with parameter adaptive decomposed images in the NSST domain and inverse NSST was applied to the fused sub-band frequency coeffi-cients to construct the final fused image. NSST coupled with PCNN decomposed images in the NSST into low-frequency coefficients and high-frequency coefficients. Specifically, the NSST is utilized to decompose the image into low and high frequency coefficients. The former are com-bined using the standard deviation from the weight region, while the latter are combined based on the NSST and PCNN. These methods focused on the AD class of MRI and PET images. DWT with transfer learning used VGG16 to determine the fusion weights for high frequencies and average low frequencies, and this is the closest to our proposed approach. Table 4 presents a comparative analysis between the outcomes of established fusion techniques and the novel approach proposed in this study.

### 5.2. Limitations

The limitations of this study include:

- The effectiveness of proposed method in extracting significant features from MRI and PET data may be limited to the specific datasets used in the study. It is important to assess its performance on a broader range of datasets to evaluate its generalizability to different imaging modalities and clinical settings.
- The proposed method should address the interpretability aspect to gain insights into the specific features extracted by the model and their clinical relevance.

## 6. Conclusions

This research demonstrates the use of deep learning techniques for the fusion of MRI PET images in AD diagnosis. By utilizing Pareto optimized model, complimentary features were captured from MRI and PET, and before the final fusion of the weight map, and extra convolution layer was added to improve the fusion process. Alignment and fusion process were improved by utilizing morphological procedures on MRI and PET images and aligning them using software tools such as Analyze 14.0 and GIMP. These techniques allowed the alignment of the images more precisely. The utilization of deep learning and image fusion methodologies in the diagnosis of AD exhibits significant potential in enhancing the precision and dependability of diagnostic protocols. The capacity to acquire and evaluate significant characteristics from multimodal imaging data may result in enhanced precision and prompt identification of AD, thereby facilitating timely intervention and treatment. Our experimental results on the ADNI dataset using various evaluation metrics, including SSIM, PSNR, MSE, and E, showed that VGG19 outperformed VGG16 and VGG11 across CN, MCI, and AD stages of AD progression. Nevertheless, additional investigation is imperative to examine alternative deep learning structures and fusion methodologies to further progress the domain of AD diagnosis. Furthermore, it is imperative to consider larger and more diverse datasets to guarantee the generalizability and robustness of the proposed methodology.

## Figures and Tables

**Table 1:** Summary of Evaluation Metrics on VGG19, VGG16, and VGG11.

*Image*	*Metrics*			
	SSIM	PSNR	MSE	E
**VGG19**				
MRI (CN)	0.680	35.43	0.15	2.850
MRI (AD)	0.802	35.93	0.12	4.510
MRI (MCI)	0.664	34.31	0.20	2.750
PET (CN)	0.669	34.18	0.28	2.830
PET (AD)	0.815	36.01	0.10	4.602
PET (MCI)	0.660	34.02	0.28	2.822
**VGG16**				
MRI (CN)	0.670	33.90	0.30	2.840
MRI (AD)	0.790	35.10	0.29	3.540
MRI (MCI)	0.600	32.90	0.40	2.620
PET (CN)	0.650	33.80	0.30	2.630
PET (AD)	0.602	33.40	0.35	2.605
PET (MCI)	0.602	32.90	0.40	2.605
**VGG11**				
MRI (CN)	0.560	33.50	0.40	1.548
MRI (AD)	0.580	33.90	0.30	1.552
MRI (MCI)	0.580	33.90	0.30	1.552
PET (CN)	0.650	33.85	0.30	1.540
PET (AD)	0.604	33.40	0.45	1.460
PET (MCI)	0.570	33.90	0.40	1.550

**Table 2:** Quality of Fused Image.

*Metrics*	*Without Transposition Layer*					*With Transposition Layer*				
	W_1_	W_2_	W_3_	W_4_	W_5_	W_1_	W_2_	W_3_	W_4_	W_5_
**MRI (CN)**										
SIMM	0.585	0.560	0.560	0.558	0.558	0.680	0.660	0.660	0.645	0.640
PSNR	29.280	29.200	29.200	29.180	29.180	35.430	35.400	35.400	35.300	35.280
MSE	0.350	0.320	0.320	0.310	0.310	0.150	0.190	0.190	0.210	0.210
E	1.950	1.900	1.900	1.890	1.890	2.850	2.760	2.760	2.680	2.650
APT (s)	0.006	0.006	0.007	0.007	0.008	0.007	0.008	0.008	0.008	0.009
**MRI (AD)**										
SIMM	0.702	0.690	0.680	0.678	0.678	0.802	0.0701	0.690	0.690	0.689
PSNR	35.850	35.800	35.700	35.700	35.700	36.930	35.930	35.800	35.800	35.800
MSE	0.180	0.180	0.170	0.170	0.170	0.120	0.130	0.180	0.180	0.180
E	4.025	3.290	3.172	3.164	3.164	4.510	4.005	3.290	3.290	3.180
APT (s)	0.006	0.007	0.007	0.008	0.008	0.007	0.008	0.008	0.009	0.010
**MRI (MCI)**										
SIMM	0.560	0.560	0.540	0.540	0.538	0.664	0.654	0.652	0.650	0.650
PSNR	29.200	29.200	29.010	29.010	29.010	34.310	34.280	34.280	34.280	34.280
MSE	0.380	0.400	0.400	0.400	0.400	0.200	0.220	0.220	0.220	0.220
E	1.900	1.900	1.700	1.700	1.690	2.750	2.740	2.740	2.740	2.740
APT (s)	0.006	0.007	0.007	0.008	0.008	0.007	0.008	0.008	0.009	0.010
**PET (CN)**										
SIMM	0.578	0.570	0.570	0.570	0.563	0.699	0.680	0.680	0.679	0.679
PSNR	29.250	29.230	29.230	29.23	29.200	35.180	34.120	34.120	34.090	34.090
MSE	0.350	0.360	0.360	0.360	0.400	0.280	0.300	0.300	0.320	0.320
E	1.998	1.908	1.908	1.908	1.899	2.830	2.730	2.730	2.690	2.690
APT (s)	0.006	0.006	0.007	0.007	0.008	0.007	0.008	0.008	0.009	0.010
**PET (AD)**										
SIMM	0.540	0.538	0.530	0.530	0.530	0.815	0.661	0.658	0.658	0.650
PSNR	29.010	29.010	28.990	28.990	28.990	36.990	35.610	35.500	35.500	35.480
MSE	0.400	0.400	0.420	0.420	0.420	0.100	0.120	0.120	0.120	0.130
E	1.700	1.652	1.650	1.650	1.650	4.602	2.745	2.748	2.748	2.740
APT (s)	0.006	0.007	0.007	0.008	0.008	0.007	0.008	0.008	0.009	0.010
**PET (MCI)**										
SIMM	0.537	0.520	0.520	0.520	0.513	0.660	0.650	0.652	0.652	0.650
PSNR	29.010	28.900	28.990	28.990	28.800	34.280	34.290	34.080	34.080	34.020
MSE	0.430	0.450	0.450	0.450	0.450	0.48	0.280	0.280	0.300	0.300
E	1.655	1.640	1.640	1.640	1.638	2.822	2.801	2.810	2.810	2.798
APT (s)	0.006	0.007	0.007	0.008	0.008	0.007	0.008	0.008	0.009	0.009

**Table 3:** Average Running Time of Pareto Optimized VGG19.

Proposed Model	Time	Hardware
With transposition convolution	0.003	GPU
Without transposition convolution (not optimized)	0.006	GPU

**Table 4:** Quantitative Measures Comparison of Proposed Method with Existing Fusion Methods.

Reference	Method	SIMM	PSNR	MSE	E
[12]	DWT with transfer learning	0.779	-	-	-
[27]	PCNN with parameter adaptive	0.7184	-	-	4.496
[28]	NSST coupled with PCNN	-	-	-	4.754
[12]	NSCT	-	-	-	2.174
Proposed Model	Pareto optimized VGG19 with transposed layer	0.802	36.93	0.12	4.510

## Data Availability

The ADNI database is available from http://adni.loni.usc.edu/ (accessed on 18 March 2023).
